# Does Faculty Follow the Recommended Structure for a New Classroom-based, Daily Formal Teaching Session for Anesthesia Residents?

**DOI:** 10.7759/cureus.818

**Published:** 2016-10-06

**Authors:** Anjum Anwar, Pedro Tanaka, Matias V Madsen, Alex Macario

**Affiliations:** 1 Department of Anesthesiology, Perioperative and Pain Medicine, Stanford University School of Medicine; 2 Department of Anesthesiology, Herlev and Gentofte Hospital, University of Copenhagen

**Keywords:** best teaching practices, adherence to a template, reinforcing learning points, employing attention grabbers in teaching, active learning

## Abstract

Background: A newly implemented 15-minute classroom-based, formal teaching session for anesthesia residents is given three times daily by the same faculty. The faculty member was provided a suggested template for the presentation. The template structure was developed by a group of residents and faculty to include best teaching practices. The goal of the current study was to measure how frequently the faculty teaching these sessions followed the template. Methods: From February 20, 2015 to February 6, 2016, a research assistant trained in education mapped a total of 48 teaching sessions to determine how frequently the teaching sessions included each of the elements in the recommended template structure. The assistant was chosen from outside the anesthesia department so as to minimize biases.

Results: It was found that 98% of the sessions used the teaching template's suggestion of using computer slides (e.g., a Powerpoint presentation). We observed that 75% of the sessions provided specific recommendations about patient care, 65% had reinforcement of learning points, 56% had a test or a quiz, 49% provided references and directions for further reading, 44% provided take-home messages, and 31% used a clinical case vignette presentation to introduce the keyword. The most common visuals were the use of a picture (38%) and a chart or a graph (35%). We also saw that 65% of the sessions had active involvement of residents. With respect to time and slide limitations mentioned in the template, we saw that 35% of the sessions finished within the recommended time limit of 15 mins and 21% had the recommended 10 or fewer slides.

Conclusion: Compliance by the faculty to the recommended structure was variable. Despite this, the sessions have been well received and have become a permanent part of the residency curriculum more than two years after their implementation.

## Introduction

A classroom-based formal lecture remains common in graduate medical education. The amount of knowledge and number of skills that anesthesiology residents need to learn increase every year as the specialty grows in scope. Since time available to learn both during direct patient care and in the classroom is limited, optimizing the education yield is a priority for every program director.

Two years ago, a new classroom-based formal teaching series was implemented when focus groups with the house staff revealed that they wanted explicit instruction of keywords for the American Board of Anesthesiology (ABA) exam [1]. The house staff also identified teaching techniques they thought the faculty should use, which was complemented by input from anesthesia faculty with expertise in medical education [2]. 

The new program consisted of a 15-minute time-limited session repeated three times daily by the same faculty member. This format enabled almost all residents to attend, given the constraints of their individual schedules. A predetermined presentation template developed by the residents and faculty was provided to the lecturers to provide uniformity for how content was delivered. This template included, for example, a brief clinical case scenario to introduce the day’s ABA keyword topic and a multiple choice question to promote interaction between the lecturer and residents. 

The best teaching practice elements in the recommended template were similar to what had been found in other studies. For example, a panel of experts found that a high-quality presenter summarizes key concepts, uses audio and/or visual aids, presents material in an organized fashion, monitors audience’s understanding of material, provides a conclusion, clearly states goals of the talk, shows enthusiasm for topic, demonstrates command of the subject matter, encourages appropriate audience interaction, and communicates the importance of the topic [3]. Also, residents prefer teaching that is directly applicable to patient care, evidence-based, short in duration, structured around clinical cases or questions, and includes active participation [4]. 

The goal of the current study was to measure how frequently the faculty teaching these sessions followed the structural elements recommended by the resident and faculty groups. These elements included using computer slides, introducing the ABA keyword with a clinical case vignette, reinforcing learning points, employing graphics or visuals, adding quizzes, providing recommendations about patient care, incorporating references/directions for further reading, eliciting active involvement of residents, listing take home messages, limiting the talk to a maximum of 10 slides, and finishing the lecture within 15 minutes. 

## Materials and methods

The Stanford Institutional Ethics Review Board deemed this study exempt from review. Since this study was considered as a program evaluation, the requirement of signed consent forms from participants was waived.

From February 20, 2015 to February 6, 2016, a research assistant trained in education attended the lectures. The assistant mapped a total of 48 sessions to one of the 10 elements in the recommended lecture structure. To address subjectivity in determining the presence or absence of some of the elements, a pilot study period was conducted where a dozen sessions were mapped and discussed with all the authors to standardize data collection. The assistant was chosen from outside the anesthesia department so as to minimize biases.

The faculty in the Department of Anesthesiology, Perioperative, and Pain Medicine selected topics for the lectures from the keyword list available at http://www.openanesthesia.org/category/aba-keywords/. Table 1 shows an excerpt from the list.

**Table 1:** ABA Keywords Sample This table is an excerpt from the keyword list available at http://www.openanesthesia.org/category/aba-keywords/.

ABA Keywords Sample	
Adrenal insufficiency: Lab finding	Cerebral aneurysm – Electrolytes
Advanced multiple sclerosis: Anesthetic drugs	Cerebral aneurysm – Transmural pressure
Aerobic vs. anaerobic glycolysis	Cerebral aneurysm clipping – Anes. management
Age-related P50	Cerebral autoregulation
Aging – CNS changes	Cerebral blood flow: Temperature effect
Aging: Cardiovascular physiology	Cerebral ischemia: Deep hypothermia
Aging: Pulmonary physiology	Cerebral vasospasm: Treatment

**Figure 1:**
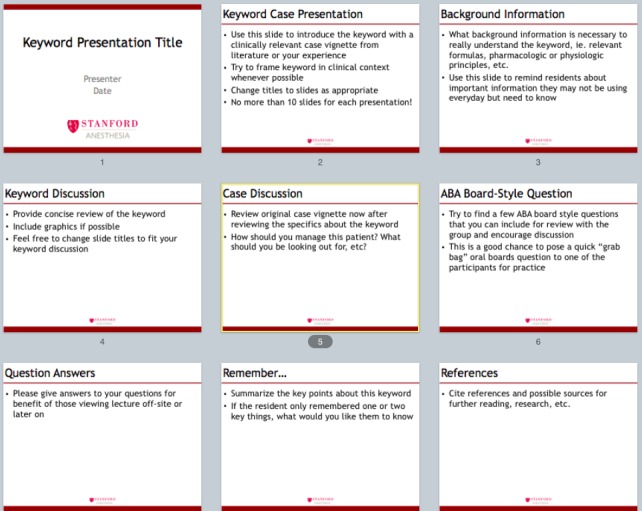
Keyword template

The following elements were included in the standardized slide template shown below in Figure 1:

-- Presenting a clinical case vignette to set up a problem-based learning experience and to introduce the keyword. The rationale is that the first moments of a presentation set the tone, capture the learners’ attention, help with the learning climate, and place the session into context.

-- Reinforcing learning points to strengthen the concepts.

-- Including specific recommendations on patient care and clinical applicability to emphasize the relevance of the topic to the learners.

-- Using graphics and visuals as attention grabbers, including the use of video clips, a cartoon, charts/graphs, or a picture. The number of images (e.g., PNG, JPEG, JPG, screenshot, or cartoon) per lecture was counted as well.

-- Using a quiz as an attention grabber to help learners interact with content.

-- Actively involving residents in the teaching session. The conceptual framework presented by Chi [5] described active, constructive, and interactive as types of overt learning activities undertaken by students. This framework generates a hypothesis that active engagements are likely to be better for learning [6]. We measured the active involvement by the number of interactions during the lecture where a learner was involved. For this study, active engagement between teacher and learner was defined as more than three engaging activities every 10 minutes of the formal teaching session. Those activities included residents asking questions, responding to questions by the instructor, or development of a group discussion [7].

-- Including references or directions for further reading. 

-- Providing explicit take-home messages at the end. 

-- Using no more than 10 slides

-- Limiting the lecture to 15 minutes

## Results

**Figure 2:**
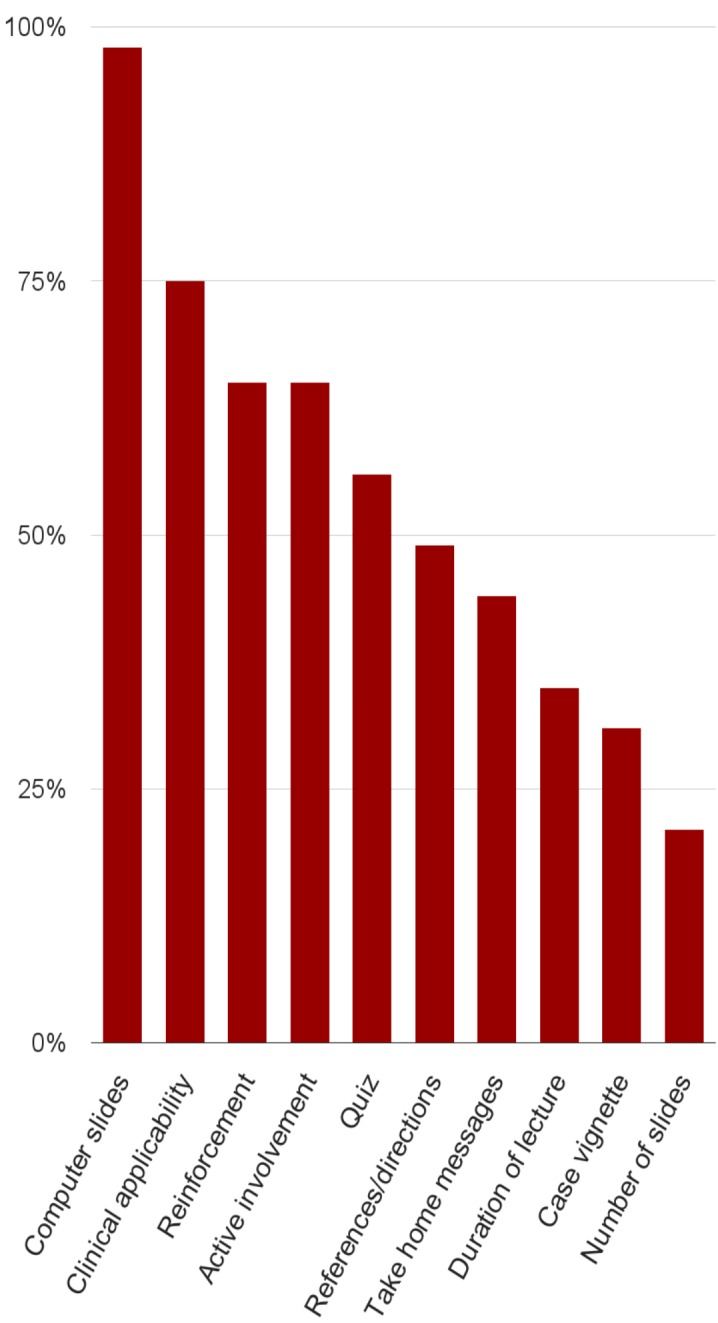
A visual display of data in Table 2

Adherence to the template ranged from a low of 21% for not exceeding the number of slides limitation to a high of 98% for using computer slides (one faculty used an erasable white board instead of computer slides). These results are shown in Table 2 and also visualized in Figure 2.

**Table 2:** Percentage of the Formal Teaching Sessions that Met Expected Structural Elements as Recommended by the Faculty and Resident Work Group

Template Element	Adherence Percentage
Computer slides (e.g., Powerpoint) presentation	98%
Clinical case vignette presentation to introduce keyword	31%
Reinforcement of learning points/repetition	65%
Specific recommendations patient care - clinical applicability	75%
Graphics and visuals:	
- video clip	4%
- cartoon	2%
- chart/graph	35%
- picture	38%
Test or quiz	56%
Active involvement of residents	65%
References/directions for further reading/studies	49%
Take home messages	44%
Lecture duration of at most 15 min	35%
A maximum of 10 slides	21%

**Figure 3:**
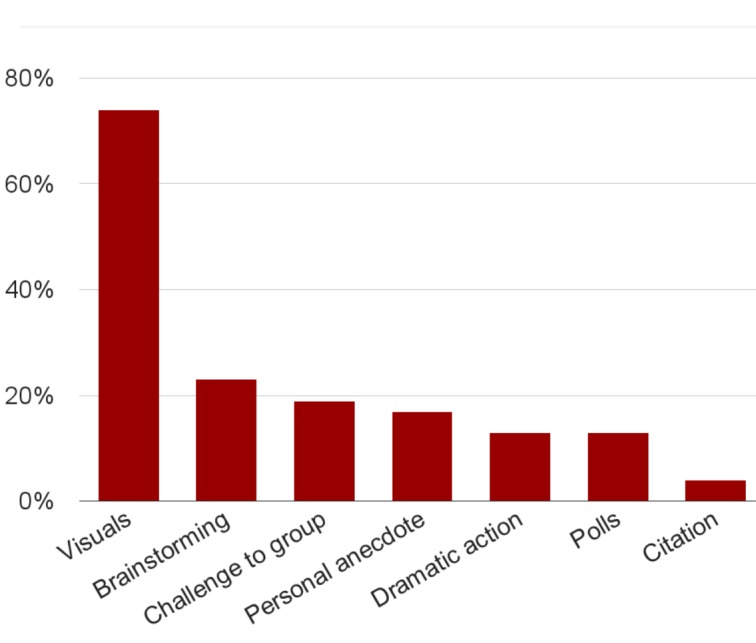
Use of attention grabbers

**Figure 4:**
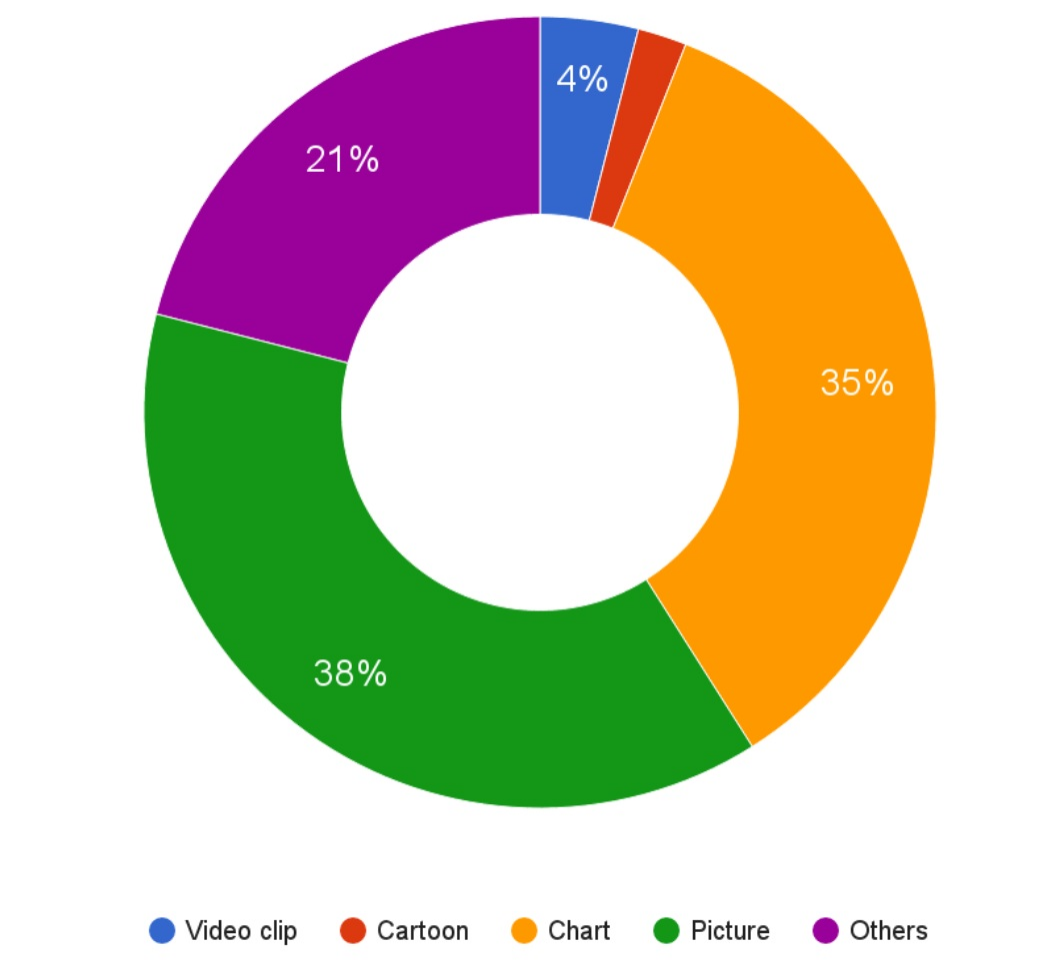
Components of graphics and visuals

The number of slides per lecture averaged 13.9 with a standard deviation (SD) of 4.2 and the number of images per lecture averaged 3.3 with an SD of 4.2. It was observed that 74% of the lectures had images. Other attention grabbers observed were brainstorming at 23% of sessions, citations at 4%, personal anecdotes at 17%, challenges to the group at 19%, dramatic action (such as imitating patients' breathing and extraordinary gesticulation) at 13%, and polls at 13%. Please see Figure 3 for attention grabbers and Figure 4 for components of visuals.

## Discussion

Compliance by the faculty to the recommended structure was variable. Despite this, the sessions have been well received and have become a permanent part of the residency curriculum more than two years after their implementation.

It is possible that the predetermined slide template structure was not explicit enough as to the required elements. Furthermore, it could be the case that not all of the elements recommended by the resident and faculty group were equally important. Efforts are underway to educate and remind faculty to take advantage of all the suggested elements.

The purpose of presenting a case vignette is to activate the resident’s prior knowledge. The conceptual framework is that prior knowledge is then built upon further as the residents at the session interact, resulting in their initial mental model to be modified and refined. As previously acquired knowledge is activated, the house staff can identify gaps in knowledge as a part of the activation–elaboration notion. Also, situational interest exists in that either the case or the test question creates a desire to find out more about the topic. This increases concentration, focuses attention, and induces a willingness to learn, motivating the resident to further inquire either with the faculty or literature until they are satisfied with their understanding [8].

Reinforcement of learning points occurred in 65% of the sessions. However, only 44% of the sessions provided explicit take home messages. This is crucial as learner engagement alternates between attention and non-attention in shorter and shorter cycles as a lecture proceeds [9]. In addition, the human cognitive system has a limited working memory that can hold five to nine pieces of information elements and can actively process up to two to four simultaneously.

We found that the faculty paid attention to mentioning the clinical applicability of the teaching session. It was observed that 75% provided specific recommendations for patient care. As for the use of attention grabbers, 74% of the lectures had one or more images. Research suggests that attention grabbers help students to learn by helping to refocus their attention [10]. Other attention grabbers included brainstorming in almost one-quarter of the sessions and a challenge to the group or a personal anecdote, each observed in almost one-fifth of sessions.

Two-thirds of the formal teaching sessions employed active learning, often with a group dialogue between faculty and residents. A study of 35-noon conferences with internal medicine residents found that 52% of the sessions had interaction with the audience (not specifically defined) and 17% included suggested reading [11]. These percentages are a bit lower than what was measured in our study, 65% and 49%, respectively. The active design elements used in our study are consistent with cognitive load theory. This framework for how people learn acknowledges that human information processing includes information presented in a visual or pictorial format and information presented in an auditory or verbal format. The lecture series structure aims to provide multiple sources of information presented in visual form (e.g., a written text and a diagram), in spoken form, and in group interaction so as not to overload the visual processor.

Given that 75% of the teaching sessions were 18 minutes or less suggests that the presenters stayed close to the recommended 15-minute time window. While only 21% of the lectures met the 10-slide limit, we observed that 76% had 16 or fewer slides. Both of these observations are encouraging as it is well-known that the audience attention span decreases significantly after 10 minutes [12].

Based on subsequent input by the trainees, a few more elements will be added to the standardized template. These include (a) explicitly defining the learning objectives to help establish expectations regarding the intended skills, attitudes, and knowledge for the learner and (b) recommending that slides have no more than seven lines (which we defined as "overly busy") [13-14]. We did relook at the slides of the 48 sessions and found that 42% of the faculty did not use any overly busy slides.

Our study had certain limitations, including that it was performed at one institution and might not be generalizable to other residencies. In addition, the teaching elements measured might not reflect all aspects of what characterizes a successful formal teaching session, such as the speakers' abilities to be engaging [15]. Furthermore, the adherence to the template might have been adversely affected by the lack of formally required instructions about being compliant with the template. It is well-known that residents also appreciate a safe learning environment, which we were not able to specifically quantify [16]. This study also did not test how the sessions affected learning retention, such as with a before-and-after written test, or a change in resident anesthesia clinical practice. 

Only a minority of the anesthesiology faculty have formal training in educating residents [17]. Most clinical faculties learn teaching techniques by primarily observing as a learner in someone else’s lecture. As a result, ongoing faculty development efforts for teaching, including in the classroom-based lecture format, are needed [18]. 

In summary, the faculty were found to more commonly follow the best teaching practices of mentioning clinical applicability, reinforcing main learning points, and active involvement of residents. The template was less helpful in ensuring some other elements, such as the presentation of a case vignette to introduce the ABA keyword.

## Conclusions

In summary, the lecture template was very helpful in ensuring that the faculty followed the best teaching practices of (a) mentioning clinical applicability, (b) reinforcing main learning points, and (c) actively involving residents. The template was less helpful in ensuring some other elements of the template, e.g., the slide limit and presentation of a case vignette to introduce the ABA keyword. We conjecture that the template’s success in ensuring the best teaching practices led to these sessions being well received. They have now become a permanent part of the residency curriculum more than two years after their implementation. ​
